# Live Dynamics of 53BP1 Foci Following Simultaneous Induction of Clustered and Dispersed DNA Damage in U2OS Cells

**DOI:** 10.3390/ijms19020519

**Published:** 2018-02-08

**Authors:** Alice Sollazzo, Beata Brzozowska, Lei Cheng, Lovisa Lundholm, Harry Scherthan, Andrzej Wojcik

**Affiliations:** 1 Centre for Radiation Protection Research, Department of Molecular Biosciences, The Wenner-Gren Institute, Stockholm University, 106 91 Stockholm, Sweden; sollazzoalice@gmail.com (A.S.); bbrzozow@fuw.edu.pl (B.B.); lei.cheng@su.se (L.C.); lovisa.lundholm@su.se (L.L.); 2 Biomedical Physics Division, Institute of Experimental Physics, Faculty of Physics, University of Warsaw, 02-093 Warsaw, Poland; 3 Bundeswehr Institute of Radiobiology, Affiliated to the University of Ulm, D-80937 Munich, Germany; scherth@web.de; 4 Institute of Biology, Jan Kochanowski University, 25-406 Kielce, Poland

**Keywords:** clustered DSB, alpha particles, X-rays, 53BP1 foci, live cell imaging

## Abstract

Cells react differently to clustered and dispersed DNA double strand breaks (DSB). Little is known about the initial reaction to simultaneous induction of DSBs with different complexities. Here, we used live cell microscopy to analyse the behaviour of 53BP1-GFP (green fluorescence protein) foci formation at DSBs induced in U2OS cells by alpha particles, X-rays or mixed beams over a 75 min period post irradiation. X-ray-induced foci rapidly increased and declined over the observation interval. After an initial increase, mixed beam-induced foci remained at a constant level over the observation interval, similarly as alpha-induced foci. The average areas of radiation-induced foci were similar for mixed beams and X-rays, being significantly smaller than those induced by alpha particles. Pixel intensities were highest for mixed beam-induced foci and showed the lowest level of variability over time as compared to foci induced by alphas and X-rays alone. Finally, mixed beam-exposed foci showed the lowest level of mobility as compared to alpha and X-ray exposure. The results suggest paralysation of chromatin around foci containing clustered DNA damage.

## 1. Introduction

Cells react to double stranded DNA damage in a highly orchestrated way [1]. The first step is damage recognition involving the NBS1-mediated activation of ATM kinase by self-phosphorylation, including a serine-1981 phosphorylation site [2]. After becoming activated, ATM has been shown to phosphorylate more than 700 substrate targets [3]. DNA damage recognition involves a complex DNA damage responsive signalling cascade that is initiated to mark the lesions and to amplify the damage signal that in turn activates the desired DNA repair pathways coordinating repair with cell cycle progression [4]. 

DNA double strand breaks (DSBs) represent particularly critical DNA lesions, because they can lead to gross chromosomal rearrangements, mutations, and cancer [5]. DSBs can be repaired by non-homologous end joining (NHEJ) during the whole cell cycle and by homologous recombination repair (HRR) during late S and G2 phase, when the sister chromatid can serve as a template [5,6]. Whichever pathway is selected, repair of lesions is preceded by histone alterations and nucleosome repositioning, as well as changes in higher-order folding of the chromatin fibre [7]. These modifications cause massive accumulation of proteins in large segments of lesion-flanking chromatin that are visible by light microscopy as nuclear foci [8]. The most relevant nuclear foci related to DSB induction which have been studied extensively are gamma H2AX [9,10] and 53BP1 foci [11,12,13]. 53BP1 foci colocalise with gamma-H2AX foci that form in the DSB-containing chromatin domains [14,15], and can thus serve as surrogate markers for DSB induction and repair [15].

Ionising radiation is a very efficient inducer of DSBs; not only in that it induces them in a dose-responsive manner [16], but it also induces qualitatively different DSBs [17], making it a valuable tool to study how cells process various forms of DSBs. Alpha particles and heavy ions, which are characterised by a high linear energy transfer (LET), ionise densely along the track and give rise to many densely spaced, complex DSBs. Complex DSBs are double-strand breaks marked by at least three single-strand breaks within 10 base pairs and other DNA damage types nearby (e.g., oxidised bases, DNA-protein crosslinks) [18,19]. X-rays and gamma radiations ionise sparsely, have a low LET, and induce predominantly simple DSBs which are randomly distributed throughout cell nuclei [17]. When nuclear foci are analysed in cells exposed to high and low LET radiation, quantitative and qualitative differences are seen: per unit dose, high LET radiation is less effective in inducing microscopically visible DSB focus numbers than low LET radiation, which reflects the fewer cell hits which suffice to reach a given dose [20,21,22]. While the majority of high LET radiation-induced foci are large, most foci induced by low LET radiation are small [20,23]. Moreover, the kinetics of focus formation is different: large high LET-induced foci form and disappear slowly, while small foci appear and disappear fast [21,22,24]. These results fit well with the notion that high LET radiation-induced complex DSBs and non-DSB clustered lesions pose a more serious problem for the DNA repair machinery as compared to simple DSBs [25,26,27,28].

The vast majority of studies aiming at analysing the relationship between radiation quality and DSB-related focus formation have been carried out with ionising radiation of a single quality. An interesting question is how cells cope with simultaneously induced localised, complex DSBs, and dispersed, simple DSBs. Such a scenario occurs when cells are exposed to a mixed beam of low and high LET radiation. Examples of such exposure scenarios are in the environment, where organisms can be exposed to a mixed field of gamma radiation (e.g., from ^226^Ra) and alpha particles (e.g., from ^222^Rn) [29]. Occupation exposures, such as during aircraft operation or space flight involve exposures to a complex mixed field of gamma radiation, neutrons, protons, and heavy ions [30,31]. Finally, during radiotherapy, patients may be exposed to gamma radiation plus neutrons, protons plus neutrons [32], or carbon ions plus neutrons [33].

Since several years, we study the cellular effects in cells exposed to a mixed beam of alpha particles and X-rays [34]. Using different endpoints, we could show that alpha particles and X-rays interact to produce more DNA damage than expected from an additive action of the two radiation types [35,36,37]. More recently, we used U2OS cells which are stably transfected with a plasmid coding for the DNA repair gene 53BP1 coupled to a gene coding for the green fluorescent protein (GFP) [12] to analyse the induction and decay of 53BP1 foci [24]. The results from analysing formaldehyde-fixed cells at various time points after radiation exposure confirmed that high and low LET radiations interact to produce more DNA damage than expected from an additive action. Moreover, they suggest an overstraining of the DNA repair system in cells exposed to mixed beams, leading to a transient retardation of complex DNA damage repair. In order to analyse in greater detail, the spatiotemporal behaviour of DSBs induced by mixed beams early after radiation exposure, we now used live cell microscopy to study selected 53BP1 foci in U2OS cells exposed to alpha particles, X-rays, and mixed beams. Cells were subjected to time lapse microscopy and images were recorded at a fixed focal plane every minute during a period of 75 min *post radiationem* (*p.r.*), analysing the dynamic behaviour of foci in time lapse series of randomly selected live cells during the chosen recording period.

## 2. Results 

The formation and decay of 53BP1-GFP foci was followed in live stably transfected U2OS cells [12]. We used live microscopy and image analysis to record the dynamics of 53BP1 foci parameters in cells after irradiation with alpha particles, X-rays, and a mixture of both during 75 min post irradiation. Image acquisition started about 10 min post exposure (*t* = 0) due to procedural steps necessary for transferring cells from the irradiation setup to the live cell chamber.

### 2.1. The Kinetics of Focus Formation and Decay

**Figure 1:**
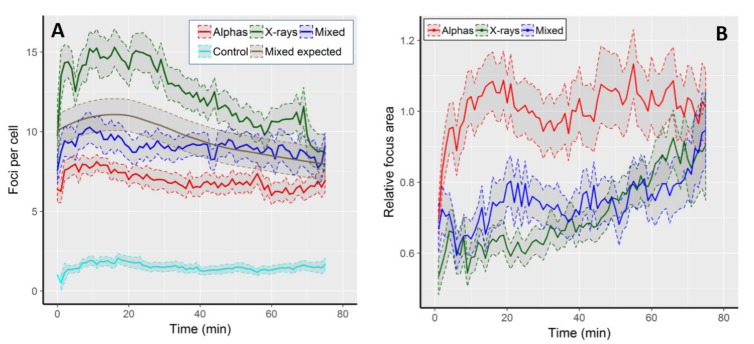
(**A**) Kinetics of 53BP1 focus frequency in control cells and following exposure to alpha particles, X-rays and mixed beams. The expected frequencies following mixed beam exposure were calculated as half of the sum of corresponding values from alpha and X-ray exposed cells, and are shown as a smooth grey curve. (**B**) Average, relative focus areas in cells exposed to alpha particles, X-rays, and mixed beams. Focus area is expressed as fraction of the respective control value. Shaded areas represent standard error of the mean.

The kinetics of focus formation is graphically shown in Figure 1A, and selected numerical values are described in Table 1. On average, we observed 1.5 (±1.5 SD) foci per cell (FPC) per control cell. All radiation qualities induced foci values well above the control level, with the level of induction and the shape of the kinetic curves being radiation-specific (Figure 1A). The highest average FPC frequency (15.3 ± 5.8) was observed in cells exposed to X-rays 16 min after the onset of image acquisition, being 10.2 times that of the control value. At 75th min, the FPC frequency dropped below the value at time 0 and was 8.7 ± 4.2, i.e., 1.8 times lower than the highest frequency. The lowest FPC frequencies were observed following alpha particle irradiation. Foci reached a peak 11 min after start of imaging (8.1 ± 2.2), being 6.9 times higher than the control and 1.2 times higher than the level of damage present at the end of imaging (6.9 ± 3.1). 

The FPC frequencies in mixed beam-exposed cells were intermediate between X-rays and alphas, with the highest level of 10.3 FPC at the 9th min after start of imaging, being 7 times higher than the control. After 75 min, the FPC frequency was 9.1 ± 2.8, being slightly lower than the initial value and 1.1 times lower than the highest frequency. Up to approximately 40 min of repair time, the FPC frequency in mixed beam-exposed cells was below the expected level (see statistical analysis for how the expected level was calculated), indicating that its shape was closer to that observed in cells exposed to alpha particles than to X-rays. The highest FPC frequency observed in X-ray-irradiated cells was significantly different (ANOVA) from the respective values scored in cells exposed to alpha particles and mixed beams. The frequencies observed in the two latter groups did not differ significantly from each other.

### 2.2. Focus Area

We next analysed the average focus areas over time. In control cells, the average focus area peaked at ~2.25 µm^2^ during the first five minutes of observation, and then gradually declined to ca. 1.4 µm^2^ at minute 75. We largely attribute the gradual decline to signal fading. In order to compensate for that, we decided to analyse the areas of radiation-induced foci as fraction of control values at the corresponding time points. The results are shown in Figure 1B. The largest foci were observed in cells exposed to alpha particles, and their size was similar to that observed in control cells (value 1 on the *Y*-axis). Following an initial increase during the first 20 min, the size of alpha-induced foci remained rather constant during the observation period. Foci induced by mixed beams and X-rays were, on average, smaller than those induced by alphas, but the difference vanished at the end of the observation period. During the initial recording period, mixed beam-induced foci were somewhat larger than those induced by X-rays, but the difference vanished after 50 min of recording time. In contrast to alpha particle-exposed cells, a steady increase in focus size was evident in both mixed beams and X-ray exposed cells. 

### 2.3. Focus Intensity Dynamics

**Figure 2:**
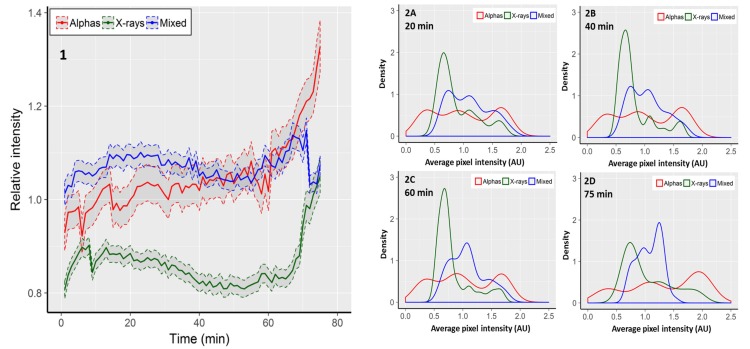
**1**: Average, relative pixel intensity per focus. Intensity is expressed as fraction of the respective control value. Shaded areas represent standard error of the mean. Distributions of foci according to average relative pixel intensity observed after 20 min (**2A**), 40 min (**2B**), 60 min (**2C**), and 75 min (**2D**). Graphs show kernel density estimations normalised to an area under the curve equal to 1. The density values correspond to relative focus frequencies. The 0 AUs shown for alpha particles are the outcome of the smoothing procedure used for density estimations.

Focus area reflects the size of a focus in two dimensions, but it does not necessarily reflect the concentration of 53BP1 molecules inside the focus. A value which is proportional to the concentration of fluorescent molecules inside a focus is the pixel intensity [38]. In order to expand the focus area measurements, average pixel intensities within single fluorescent foci were recorded. Due to repeated fluorescent excitation of cells, the intensity of pixels faded with time. This was detected as a linear decrease of the average pixel intensity in control cells from 139.3 arbitrary fluorescent units (AU) at time 0 to 104.6 AU at the 75th minute. In order to take the observed fading into account, the results observed in irradiated cells are presented as fractions of the respective average pixel intensity observed in control cells (Figure 2, panel 1). Cells exposed to mixed beams showed a relatively constant level of pixel intensity. During the first 50 min of recording, the intensity level was higher than in cells exposed to alpha particles and X-rays. However, after ~60 min, foci induced by alpha particles became brighter than those induced by mixed beams, and for the remaining 15 min showed a steadily increasing brightness (Figure 2). The lowest level of pixel intensity was observed in cells exposed to X-rays. Similarly as for mixed beam exposure, foci induced by X-rays showed a relatively constant level of pixel intensity until minute 50, and became increasingly brighter thereafter. The CV values for alpha particles, X-rays, and mixed beams were, respectively, 0.071, 0.058, and 0.025, substantiating the observation that during the whole observation period, cells exposed to mixed beams showed the lowest variability of pixel intensity. 

Next, we analysed the distributions of average, relative pixel intensities per focus. The results are shown in Figure 2, panels 2A–2D, in the form of kernel density estimations. Foci induced by alpha particles showed the broadest and most even distribution of pixel intensities, without any distinct intensity peaks. A gradual shift towards a higher intensity was observed with increasing recording time. Moreover, the maximal intensity increased from ca 2.1 at minute 20, to 2.5 at minute 75. Foci induced by X-rays showed a distinct intensity peak in the range of 0.5–1.0 AU. The peak was gradually reduced, but was still clearly discernible at minute 75. The maximal focus intensity also increased from ca. 1.9 at minute 20 to 2.2 at minute 75. Foci induced by mixed beams showed the most uniform distribution with a double-peaked plateau between ca. 0.6 and 1.5 AU at minute 20. The right peak gradually increased until minute 75, however, the maximal intensity decreased from ca. 2.0 at minute 20, to ca. 1.6 at minute 75. In summary, mixed beam-induced foci showed the lowest degree of variability and dynamics of pixel intensity throughout the observation period.

### 2.4. Focus Mobility

**Figure 3:**
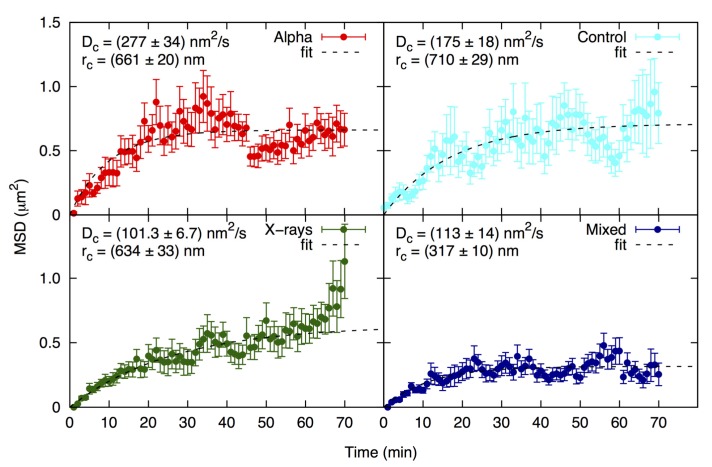
Mean square displacement (*MSD*) analysis of alpha (red), X-ray (green), mixed beam (dark blue)-induced foci, and control (light blue) foci. *D_c_* = diffusion coefficient; *r_c_* = radius of constraint.

The identification of individual foci by the image analysis software allowed to follow their mobility on the focal plane of the cell nucleus. The results of the analysis are shown in Figure 3 as mean square displacement (*MSD*) analysis, along with the values for *D*_c_ (diffusion coefficient) and *r*_c_ (radius of constraint). *MSD* is the square of the path length, *D_c_* is a measure of focus mobility, and *r_c_* is the radius of a sphere within which the focus moved (see Figure 5 for a graphic example of focus movement). Foci in all cell groups showed a typical pattern of random walk in a confined space, as was demonstrated in other cell systems [39]. The treatments can be ranked according to the highest degree of focus movement in the following order: alpha particles > controls > X-rays > mixed beams, revealing a paralyzing effect of mixed irradiation on focus mobility. All *D_c_* and *r_c_* values significantly differed from each other (one-way ANOVA), except for *D_c_* for X-rays vs. mixed beams. 

### 2.5. Focus Merging and Splitting

**Figure 4:**
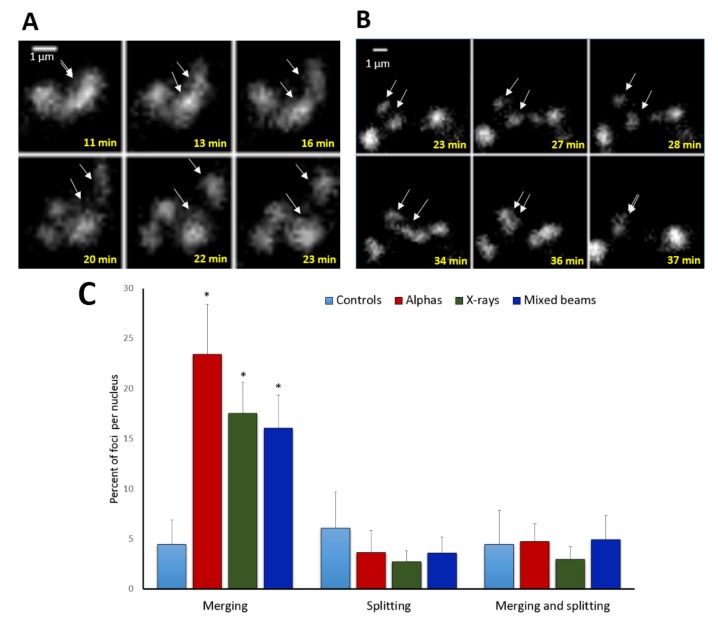
Examples of focus splitting (**A**), merging (**B**), and statistics (**C**). Arrows mark foci that split or merge. (**A**) foci induced by X-rays, (**B**) foci induced by alpha particles; (**C**) frequency of focus merging and splitting events per nucleus. “Merging and splitting” refers to foci that exhibited both behaviours. White arrows point at sites of foci splitting and merging. Error bars: standard deviation. * significant differences to control (one way ANOVA).

In addition to focus movement, a merging and splitting behaviour of foci was noted. Examples of foci splitting and merging are shown in Figure 4A,B. Relative numbers of foci that split or merged are shown in Figure 4C. Generally, many more foci merged than did split. The highest level of merging was observed in cells exposed to alpha particles (24% of foci per nucleus), followed by X-rays (17%) and mixed beams (16%), with the differences being insignificant and in agreement with a low chromatin mobility after mixed beam irradiation. Less than 5% of control foci merged. The level of splitting or both merging and splitting was similar for all IR qualities and the control, and oscillated around 5%. 

## 3. Discussion

The aim of this study was to investigate the dynamics of radiation-induced individual 53BP1-GFP foci in live cells following exposure to high LET alpha particles, low LET X-rays and a mixed beam of both. To this end, we applied live microscopy and semiautomatic image analysis to follow the behaviour of foci at a fixed optical plane in nuclei using image recording at a frequency of 1 image per min during 75 min post irradiation. The various types of ionising radiation were used as tools to study the reaction of cells to clustered DSB (alpha particles), dispersed simple DSB (X-rays), and a combination of both. 

The major difference in biological action of alpha particles and X-rays is the mode of energy deposition within a target volume. Irradiation of cells with a unit dose of radiation means that the average amount of energy deposited inside a cell nucleus is the same for any type of ionising radiation, albeit, with a different spatial distribution. While X-rays deposit the energy sparsely and more or less evenly inside a cell nucleus, alpha particles deposit the energy densely, along discrete tracks. The consequence, with respect to DSBs, is that alpha particles induce a higher average number of DSBs per cell nucleus and unit dose [40]. Alpha particle-induced foci are on average larger than those induced by X-rays [22]. Per unit dose, fewer cells are hit by alpha particles than X-rays, and alpha particles induce a higher degree of complex DSB than X-rays [17,22]. We calculated that a dose of 1 Gy corresponds on average to 5 alpha tracks per cell nucleus, while 1 Gy of X-rays corresponds, on average, to 2000 photon tracks per cell nucleus (unpublished results). Consequently, a 50% mixed beam scenario corresponds, on average, to 2.5 tracks from alpha particles, and 1000 tracks from X-ray photons. 

The obtained results showed that foci induced by mixed beams seldom behaved as expected, assuming that they should reflect an average response to both clustered and dispersed DSBs coming from, respectively, alpha particles and X-rays. In short, mixed beam-induced foci (1) lacked a fast increase and decline of their frequency, such as was observed following irradiation with X-rays alone, and in this respect, were similar to alpha particles alone; (2) showed focus areas similar to those induced by X-rays, and significantly smaller than those induced by alpha particles; (3) showed a high mean pixel intensity which was less variable than that induced by alpha particles and X-rays alone; (4) showed a distinctly lower diffusion than for alpha particle and X-ray-induced foci. 

During the first 40 min of observation, the highest frequency of 53BP1-GFP foci was observed in cells exposed to X-rays, followed by mixed beams and alpha particles. The high frequency of X-ray induced foci was expected in light of the difference in the distribution of tracks between the radiation types, and the fact that DSBs which are induced in proximity to each other (as is the case with alpha particles) can form a single focus [41,42].

The high effectiveness of X-rays as compared to alpha particles in inducing damage foci was also reported by others [22], and by us in an earlier investigation, where we analysed 53BP1-GFP foci in fixed U2OS cells [24]. In that earlier investigation, we found that mixed beams induced the same average frequency of foci as X-rays, and we interpreted this finding as evidence for a synergistic action of alpha particles and X-rays. In the present investigation of live cells, we observed a lower than expected frequency of foci in cells exposed to mixed beams, which is most probably due to a different microscopic setup. While in the earlier investigation we analysed fixed cell nuclei and detected foci in the entire nucleus, the present live cell investigation was based on repetitive images of foci at a single focal plane. The repeated light exposures resulted in a 25% signal loss over the 75 min recording interval. Furthermore, live cells were analysed at a single, fixed optical plane, so foci lying above or below the plane were missed. In contrast, the previous analysis of fixed cells [24] permitted a more complete scoring of focus numbers, because fixed cells are flattened during the fixation process. Indeed, for all radiation types, focus frequencies in live cells observed at a fixed optical plane were distinctly lower than the corresponding frequencies observed in fixed cells [24]. Therefore, it appears that the focus frequencies analysed in live cells at a single optical plane cannot be directly compared to the numbers obtained in fixed cells.

Another intriguing result is that mixed beams induced particularly many low intensity foci which are believed to represent simple DSBs [22]. Here, a remarkable observation is the apparent lack of focus decay both in cells exposed to alpha particles and mixed beams during the first 75 min of observation relative to X-rays. High LET radiation induces many complex DSBs, and these are repaired slowly [43]. By contrast, X-ray-induced foci showed a fast increase and decline, and it is believed that X-ray-induced foci contain simple DSBs which are repaired quickly [22]. Hence, the lack of focus decay reported here in cells exposed to alpha particles is not surprising. However, despite the fact that the mixed beam consisted to 50% of X-rays, no decay of foci was detected during the 75 min of observation. The frequency of foci induced by mixed beams was higher than that of alpha particles, which obviously must be due to the action of low LET X-rays scattered inside the nuclear volume. But why were there no foci that showed a fast decay indicative of fast DNA repair, which is typical for X-rays? A possible explanation could be the migration of X-ray induced DSBs to sites of repair of alpha particle-induced clustered DSBs. 

It is believed that DSBs can migrate and cluster to form repair centres [39,42,44,45]. If, in mixed beam-exposed cells, X-ray-induced DSBs migrated to sites of clustered DSBs induced by alpha particles, then the mixed beam-induced foci should be particularly large. This, however, was not observed at the level of focus area. On the contrary, at most time points, foci induced by mixed beams had a similar mean area as foci induced by X-rays, this being in contrast to alpha particle-induced foci, which showed a distinctly higher mean area, at least during the first 60 min. However, foci induced by mixed beams showed the highest average pixel intensity during the first 40 min of exposure, indicating a strong concentration of 53BP1 molecules. Why did this not lead to enhanced focus areas? Here, it must be recalled that 53BP1 does not penetrate heterochromatin [46,47], so perhaps chromatin at mixed beam-induced foci shows a particular high degree of relaxation and, consequently, a high concentration of 53BP1. Additionally, Neumaier et al. [42] observed that, following increasing doses of X-rays, 53BP1 focus intensities increased with the dose without a concomitant increase of focus sizes, suggesting that 53BP1 protein molecules which accumulate at sites of multiple DSBs are compacted within the focus structure. It is also interesting to note that mixed beam-induced foci showed the lowest variability of pixel intensity over time, corroborating the idea of a qualitative difference between these foci, and those induced by alpha particles and X-rays alone. Neumaier et al. [42] suggested that enhanced migration of DSBs to DNA repair centres will lead to the formation of complex chromosomal aberrations (defined as chromosomal exchanges involving three or more breaks in two or more chromosomes [48]). In accordance with this, we have indeed observed a high frequency of complex aberrations in human peripheral blood lymphocytes exposed to the same mixed beams of alpha particles and X-rays as used in the present study [36]. This finding again supports the assumption that mixed beam-induced foci contain complex plus simple DSBs that may merge to form repair centres. 

With respect to the spatial distribution of DSBs induced by alpha particles and X-rays, it was shown that charged particles, such as alphas, ionise densely inside the core of a track [49]. Delta electrons are ejected from the core, forming a penumbra which is similar in its radiation chemistry characteristics to a photon-irradiated target [50]. Hence, in a mixed beam scenario, the impact of X-rays on ionisation density inside the core of a track is negligible, and it can be expected that an interaction of alpha particles and X-rays, which may lead to an increase of ionisation density resulting in increased complexity of DSBs, occurs preferentially in the penumbra of the alpha tracks. We are currently carrying out Monte Carlo simulations to better understand the interaction mechanisms of alpha particles and X-rays in inducing DNA damage. 

A further remarkable observation was that foci induced by mixed beams showed a lower degree of mobility than control foci, and those induced by alpha particles and X-rays. The mobility of damaged chromatin regions within the nucleus is currently in the focus of research [7,45,51,52]. It was reported that foci induced by high LET radiation show a high mobility [53,54]. This fits well with our results obtained in cells exposed to alpha particles, not only with respect to mobility of foci, but also to merging of foci. But why did foci induced by mixed beams show a reduced mobility? It was shown that subdiffusion is a consequence of crowding-induced alteration of viscoelasticity [55]. It is possible that the low mobility of detectable, mixed beam-induced foci is due to the presence of repair proteins clustering at dispersed, non-DSB DNA damage, induced by X-rays present in the mixed beam. 

In the present study, we exclusively analysed 53BP1-GFP focus formation in live cells because of the availability of the transfected cell line, and in order to obtain insights into live cell DSB focus dynamics in comparison to our previous fixed cell approach using the same cell line. Further analyses will address the interesting question regarding colocalisation of 53BP1 with other proteins of the DNA damage response pathway and/or proteins involved in repair of non-DSB lesions, such as oxidatively-clustered DNA lesions (OCDLs) [19]. It is possible that OCDLs are preferentially formed by combined exposure to high LET alpha particles, which mainly damage the DNA in a direct way inside the core of an ion track [50], and low LET X-rays, which mainly damage the DNA indirectly, via free radicals. To this end, Georgakilas and colleagues have recently suggested an analysis parameter, termed P_clc_, which provides an estimate of the relative localisation of non-DSB repair proteins on a DSB focus area, compared to its localisation in the rest of the cell nucleus, representing the endogenous level of the non-DSB related repair proteins [28].

Because the dose rate of alpha particles (0.24 Gy/min plus 25 mGy/min beta) was higher than that of X-rays (0.052 Gy/min in the top table position and 0.068 Gy/min in the bottom table position), the exposure to alphas was shorter than to X-rays by nearly 10 min, and to mixed beams by nearly 4 min. In order to be consistent with our earlier studies, we always started the combined exposure simultaneously [21,36,37]. The low dose rate of X-rays could mean that some DNA lesions were repaired already during the irradiation by fast non-homologous end joining (NHEJ) repair [1], leading to a reduced level of initial damage at the start of imaging, as compared to alpha particles and mixed beams. Such an effect was, however, not observed, with X-ray-induced foci, showing the typical increase and decline of focus frequencies during the first 30 min post exposure. Thus, we assume that the differences in dose rates between the different radiation types did not have a confounding impact on the interpretation of the results.

## 4. Materials and Methods 

### 4.1. Cell Culture and Cell Line Authentication

All experiments were carried out with human osteosarcoma U2OS cells which were stably transfected with a plasmid coding for 53BP1-GFP. The transfected cells were kindly provided by Dr. Claudia Lukas from the Danish Cancer Society, Copenhagen, and their characteristics are described elsewhere [12]. Cell line authentication was carried out by regular testing for the phenotype of 53BP1-GFP focus expression. A high level of expression was assured by culturing cells in a selection medium containing geneticin to eliminate cells that lost the phenotype. The complete culture medium was composed of Dulbecco Modified Eagle Medium (Sigma-Aldrich, D6046, Stockholm, Sweden) supplemented with 10% bovine calf serum (Thermo Scientific, cat.no SH30072.3, Stockholm, Sweden) and 400 µg/mL geneticin G418 (Sigma-Aldrich, AI720). Cells were kept in a 5% CO_2_ humidified 37 °C incubator. Before irradiation, cells were grown on round glass coverslips.

### 4.2. Irradiation

Exponentially growing cells were irradiated on round glass coverslips (diameter: 25 mm) with 1 Gy of alpha particles, 1 Gy X-rays, or a combination of 0.5 Gy alpha particles and 0.5 Gy X-rays. The latter is referred to as the mixed beam. Irradiations were carried out at room temperature using a dedicated facility which is installed at the Center for Radiation Protection Research at the Department of Molecular Bioscience the Wenner-Gren Institute, Stockholm University. Details of the facility are described in [34,35]. In brief, it consists of an X-ray irradiator installed below an ^241^Am alpha source (activity: 50.0 ± 7.5 MBq) [34,35]. The alpha source delivers alpha particles at a fluence of 23,800 ± 4600 particles per second. The resulting dose rate is 0.265 Gy/min. Since the alpha particles are not collimated, cells are hit from all possible angles. In consequence, particles of various energies enter the cells and ionise with a span of LET values that vary in the range of 100 to 172 keV µm^−1^ at the entrance of the cell layer, and of 100 to 238 keV µm^−1^ as the particles penetrate the cells. An alpha dose of 0.2 Gy corresponds, on average, to one hit per cell. Ten percent of the alpha dose comes from beta radiation. 

For irradiation, round coverslips fitting into the custom-made live cell chambers (see below) were placed with cells facing upwards on specific polyamide disks, 155 mm in diameter, custom-constructed in the Institute for Energy-JRC, Petten, the Netherlands and covered with a 1.5 µm thick, bilaterally oriented Mylar foil lid (Goodfellow, Cambridge, UK). The irradiation disks were positioned on a movable shelf which, in the default bottom position, is ca. 10 cm below the alpha source. Alpha irradiation was initiated by moving the disk upwards, close to the source (table top position). 

X-ray irradiation was carried out with a SMART 200 X-ray tube (Yxlon, Hamburg, Germany), operating at 190 kV and 4.0 mA, aluminium filtering) which was positioned ca. 30 cm below the alpha irradiator. The X-ray beam had an energy peak at 80 keV [56]. The dose rate was 0.068 Gy/min at the bottom disk position and 0.052 Gy/min at the top disk position. 

Mixed beam irradiation consisted of a simultaneous exposure to 0.5 Gy alpha and 0.5 Gy X-rays. Combined exposure to alpha particles and X-rays always started simultaneously, with the cells positioned close to the alpha source (table top position) and the X-ray source on. However, due to differences in the dose rate between the two radiation qualities, the alpha irradiation was stopped 5 min before the X-rays. The following irradiation times were applied: 3 min and 46 s for 1 Gy of alpha particles, 14 min and 21 s for 1 Gy of X-rays, and 7 min and 50 s for 1 Gy of mixed beam (1 min and 53 s for alpha radiation and 7 min and 50 s for X-rays). 

A dose of 1 Gy alpha radiation corresponds on average to 5 alpha tracks per cell nucleus, while 1 Gy of X-rays corresponds on average to 2000 photon tracks per cell nucleus. 

### 4.3. Live Cell Imaging

Immediately after irradiation, coverslips with irradiated cells were placed in a pre-warmed stainless steel live cell chamber as described by [57]. The chamber, the size of a standard microscopic slide, had a bottomless well, in which the round coverslip can be positioned and sealed by a Sykes-Moore gasket (O-ring and a plastic nut). This well was then filled with pre-warmed growth medium supplemented with sodium pyruvate and 10 mM HEPES (Applied Chem GmbH, A3268, Hamburg, Germany) for pH stability. A coverslip was loosely placed on top of the filled well to prevent evaporation of the medium. The live cell chamber was assembled immediately after irradiation, a process which took ca. 10 min. Time-lapse microscopy started thereafter, and was performed at 37 °C inside a self-constructed temperature-controlled microscope environmental chamber mounted on a Carl Zeiss Axiovert 200 inverted microscope (Jena, Germany). A group of cells was randomly selected, and images were taken once every minute for 75 min, using a 63× oil immersion objective. GFP fluorescence was induced by short (200 ms) periods of illumination with 400 nm light of a Polychrome IV monochromator (TILL Photonics, Gräfelfing, Germany). Images were acquired with the optical plane at the nuclear equator using the TILL Photonics Imaging System (TILLvisION v3.3, Gräfelfing, Germany) installed at the inverted Zeiss microscope [58]. Experiments were repeated until, for each radiation quality, time lapse movies of 25 cells were recorded. Each time lapse movie encompassed 75 images recorded at a speed of 1 image per minute. Thirty non-irradiated control nuclei were recorded at the same image frequency and for the same period of time. Per nucleus, all visible foci at a fixed nuclear plane were analysed on the 75 recorded images. The acquisition of a complete set of movies for all radiation qualities and controls required at least 10 independent experiments. The image acquisition time was limited to 75 min, because maintaining cells in the stainless steel live cell chamber for longer periods of time resulted in morphological changes of cell shape, possibly due to evaporation of the cell culture medium.

### 4.4. Image Analysis

**Figure 5:**
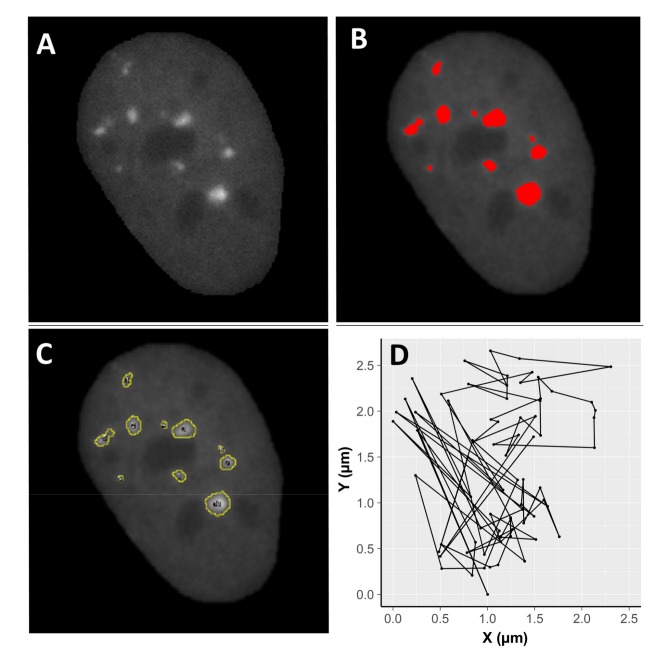
Example of image processing using Fiji. A cell nucleus after selection and filtering (**A**), focus recognition (**B**), and identification (**C**). Image (**D**) demonstrates two dimensional (X,Y) focus movement inside a nucleus stabilized by rigid body transformation (see Section 4. Materials and Methods). A total of 25 irradiated nuclei per radiation type and 30 control nuclei were analysed.

Images were analysed with the Fiji image processing package (Laboratory for Optical and Computational Instrumentation, University of Wisconsin-Madison, Madison, WI, USA). For every picture, cell nuclei were isolated by drawing a region of interest (ROI) around the nucleus and clearing the background outside the selected area (Figure 5). Movements of cells were stabilized using rigid body transformation (Macro StackReg by Philippe The’venaz, Biomedical Imaging Group, Swiss Federal Institute of Technology, Lausanne, Switzerland), which corrects for translational and rotational motion of the whole nucleus. Thus, the resulting motion of foci is independent of nuclear motion. After filtering (smoothing) and application of the appropriate threshold, information about nuclear area, as well as focus frequency per cell, focus area, and mean pixel intensity per focus, were gathered. Twenty-five nuclei per radiation type and 30 control nuclei were processed for an incubation time of 75 min. Moreover, movement of selected foci was monitored. To this end, ROIs were drawn around individual, randomly chosen foci. The mean intensity, area and coordinates of the centre of mass of each focus in the ROI were determined. Data relative to merging foci or external foci migrating into the ROI were removed after visual inspection. One hundred and fifty foci were analysed in cells exposed to alpha particles, 189 in cells exposed to X-rays, and 169 in cells exposed to mixed beams. 

Events caused by interaction between foci were quantified by visual inspection. Interacting events were categorized according to behaviour of foci during the imaging time into (1) splitting: a focus splits into two or three smaller foci; (2) merging: two or three foci merge into one bigger focus; and (3) merging and splitting: two or three foci that merged for some seconds and split again. 

All data were first stored as separate text files, and then analysed using the R statistical software (Windows version, Institute for Statistics and Mathematics, Wirtschaftsuniversität Wien, Vienna, Austria) [59].

### 4.5. Statistical Analysis

All statistical analyses were performed with the Gnuplot 5.0 (Available online: www.gnuplot.info), the software R or SigmaStat 3.5 (Systat Software GmbH, Erkrath, Germany). Differences were tested with one-way ANOVA or *chi-square* test with a 2 × 2 contingency table. The value *p* < 0.05 was considered statistically significant. 

Focus displacement was fitted by the subdiffusion model [52], also known as random walk in a confined space [39], using the following equation in R:

(1) $$MSD\left(∆t\right)=r_{c}^{2}\;\left\{1-\exp\left(-\frac{2dD_{c}∆t}{r_{c}^{2}}\right)\right\},$$

where *MSD* = the mean square displacement; *D*_c_ = the diffusion coefficient; *r*_c_ = the diffusion radius; Δ*t* = the time interval; and *d* = the dimension of the analysed space (equal 2).

Distributions of focus intensities were fitted by Gaussian kernel diffusion estimates using the following equation in R:

(2) $$f_{h}\left(x\right)=\frac{1}{nh}\overset{n}{\underset{i=1}{\sum }}K(\frac{x\;-\;Xi}{h}),\;\;K\left(x\right)=\frac{1}{\sqrt{2\pi }}\exp\left(-\frac{x^{2}}{2}\right),$$

where *f_h_* = the probability density function; *h* = the bandwidth parameter; *K* = the kernel function; *x* = argument of the function (focus intensity); and *n* = size of the sample. 

The expected frequencies of foci in cells exposed to mixed beams were calculated as half of the sum of corresponding values from alpha and X-ray exposed cells. The calculations were performed using fitted focus frequencies (fitting was carried out with R). The objective of using fitted values was smooth, expected focus frequency kinetics, facilitating a visual comparison to the observed values. 

Dynamics of mean pixel intensities per focus in cells exposed to alpha particles, X-rays, and mixed beams were compared by calculating coefficients of variation (CV). To this end, the mean pixel intensities and standard deviations were calculated for complete measurement series from minute 1 to minute 75. 

## 5. Conclusions

In conclusion, our results suggest that cells react to a simultaneous induction of clustered and dispersed DNA damage by concentrating the 53BP1 protein in particular foci, possibly preferentially at sites of clustered DNA damage that are formed early during the irradiation process. Moreover, mixed beam-induced foci show a low degree of mobility, possibly contributing to augmented misrepair of damage, especially at clustered DSBs. These results support our earlier observation of a high frequency of complex chromosomal aberrations in cells exposed to mixed beams. With respect to the carcinogenic effect of radiation, the results demonstrate that cells exposed to a mixed beam of high and low LET radiation process the damage in a manner not predicted by assuming a simple additive action of both radiation types. 

## Figures and Tables

**Table 1:** Numerical values describing the initial (0 min, imaging start), maximum (highest), and final (75 min) frequencies of 53BP1 foci per cell (FPC). The expected FPC values following mixed beam exposure were calculated as mean of corresponding values from alpha and X-ray exposed cells. Error values represent standard deviation. *: significantly different from alpha particles (one-way ANOVA).

Radiation Type	Initial FPC	Highest FPC (@ Time Point)	Final FPC	Max. Focus Increase ^1^	Max. Focus Reduction ^2^
**Control**	1.5 ± 1.5	1.5 ± 1.5	1.5 ± 1.5	-	-
**Alpha particles**	6.4 ± 3.1	8.1 ± 2.2 (11 min)	6.9 ± 3.1	5.4	1.2
**X-rays**	9.7 ± 4.3	15.3 ± 5.8 * (16 min)	8.7 ± 4.2	10.2	1.8
**Mixed beams**	7.6 ± 3.4	10.3 ± 4.8 (9 min)	9.1 ± 2.8	6.8	1.1
**Expected**	8.05	11.7 (15 min)	7.8	7.8	1.3

^1^ highest FPC divided by control FPC; ^2^ difference between highest FPC and final FPC.

## Abbreviations

DSB	DNA double strand breaks
FPC	frequency per cell
GFP	green fluorescence protein
HRR	homologous recombination repair
LET	linear energy transfer
NHEJ	non-homologous end joining
*p.r.*	*post radiationem*
SD	standard deviation

## References

[1] Jeggo P.A., Lobrich M. (2006). Radiation-induced DNA damage responses. Radiat. Prot. Dosim..

[2] Blackford A.N., Jackson S.P. (2017). ATM, ATR, and DNA-PK: The Trinity at the Heart of the DNA Damage Response. Mol. Cell.

[3] Matsuoka S., Ballif B.A., Smogorzewska A., McDonald E.R., Hurov K.E., Luo J., Bakalarski C.E., Zhao Z., Solimini N., Lerenthal Y. (2007). ATM and ATR substrate analysis reveals extensive protein networks responsive to DNA damage. Science.

[4] Dantuma N.P., van Attikum H. (2016). Spatiotemporal regulation of posttranslational modifications in the DNA damage response. EMBO J..

[5] Schipler A., Iliakis G. (2013). DNA double-strand-break complexity levels and their possible contributions to the probability for error-prone processing and repair pathway choice. Nucleic Acids Res..

[6] Branzei D., Foiani M. (2008). Regulation of DNA repair throughout the cell cycle. Nat. Rev. Mol. Cell Biol..

[7] Panier S., Boulton S.J. (2014). Double-strand break repair: 53BP1 comes into focus. Nat. Rev. Mol. Cell Biol..

[8] Lukas J., Lukas C., Bartek J. (2011). More than just a focus: The chromatin response to DNA damage and its role in genome integrity maintenance. Nat. Cell Biol..

[9] Rogakou E.P., Boon C., Redon C., Bonner W.M. (1999). Megabase chromatin domains involved in DNA double-strand breaks in vivo. J. Cell Biol..

[10] Sedelnikova O.A., Pilch D.R., Redon C., Bonner W.M. (2003). Histone H2AX in DNA damage and repair. Cancer Biol. Ther..

[11] Schultz L.B., Chehab N.H., Malikzay A., Halazonetis T.D. (2000). p53 binding protein 1 (53BP1) is an early participant in the cellular response to DNA double-strand breaks. J. Cell Biol..

[12] Bekker-Jensen S., Lukas C., Melander F., Bartek J., Lukas J. (2005). Dynamic assembly and sustained retention of 53BP1 at the sites of DNA damage are controlled by Mdc1/NFBD1. J. Cell Biol..

[13] Lottersberger F., Karssemeijer R.A., Dimitrova N., de Lange T. (2015). 53BP1 and the LINC Complex Promote Microtubule-Dependent DSB Mobility and DNA Repair. Cell.

[14] Markova E., Schultz N., Belyaev I.Y. (2007). Kinetics and dose-response of residual 53BP1/gamma-H2AX foci: Co-localization, relationship with DSB repair and clonogenic survival. Int. J. Radiat. Biol..

[15] Martin O.A., Ivashkevich A., Choo S., Woodbine L., Jeggo P.A., Martin R.F., Lobachevsky P. (2013). Statistical analysis of kinetics, distribution and co-localisation of DNA repair foci in irradiated cells: Cell cycle effect and implications for prediction of radiosensitivity. DNA Repair.

[16] Erixon K., Cedervall B. (1995). Linear induction of DNA double-strand breakage with X-ray dose, as determined from DNA fragment size distribution. Radiat. Res..

[17] Goodhead D.T. (2006). Energy deposition stochastics and track structure: What about the target?. Radiat. Prot. Dosim..

[18] Nikjoo H., O’Neill P., Goodhead D.T., Terrissol M. (1997). Computational modelling of low-energy electron-induced DNA damage by early physical and chemical events. Int. J. Radiat. Biol..

[19] Mavragani I.V., Nikitaki Z., Souli M.P., Aziz A., Nowsheen S., Aziz K., Rogakou E., Georgakilas A.G. (2017). Complex DNA Damage: A Route to Radiation-Induced Genomic Instability and Carcinogenesis. Cancers.

[20] Leatherbarrow E.L., Harper J.V., Cucinotta F.A., O’Neill P. (2006). Induction and quantification of gamma-H2AX foci following low and high LET-irradiation. Int. J. Radiat. Biol..

[21] Staaf E., Brehwens K., Haghdoost S., Czub J., Wojcik A. (2012). Gamma-H2AX foci in cells exposed to a mixed beam of X-rays and alpha particles. Genome Integr..

[22] Antonelli F., Campa A., Esposito G., Giardullo P., Belli M., Dini V., Meschini S., Simone G., Sorrentino E., Gerardi S. (2015). Induction and Repair of DNA DSB as Revealed by H2AX Phosphorylation Foci in Human Fibroblasts Exposed to Low- and High-LET Radiation: Relationship with Early and Delayed Reproductive Cell Death. Radiat. Res..

[23] Costes S.V., Boissiere A., Ravani S., Romano R., Parvin B., Barcellos-Hoff M.H. (2006). Imaging features that discriminate between foci induced by high- and low-LET radiation in human fibroblasts. Radiat. Res..

[24] Sollazzo A., Brzozowska B., Cheng L., Lundholm L., Haghdoost S., Scherthan H., Wojcik A. (2017). Alpha particles and X-rays interact in inducing DNA damage in U2OS cells. Radiat. Res..

[25] Hada M., Georgakilas A.G. (2008). Formation of clustered DNA damage after high-LET irradiation: A review. J. Radiat. Res..

[26] Meyer B., Voss K.O., Tobias F., Jakob B., Durante M., Taucher-Scholz G. (2013). Clustered DNA damage induces pan-nuclear H2AX phosphorylation mediated by ATM and DNA-PK. Nucleic Acids Res..

[27] Lorat Y., Timm S., Jakob B., Taucher-Scholz G., Rube C.E. (2016). Clustered double-strand breaks in heterochromatin perturb DNA repair after high linear energy transfer irradiation. Radiother. Oncol..

[28] Nikitaki Z., Nikolov V., Mavragani I.V., Plante I., Emfietzoglou D., Iliakis G., Georgakilas A.G. (2016). Non-DSB clustered DNA lesions. Does theory colocalize with the experiment?. Radiat. Phys. Chem..

[29] Hendry J.H., Simon S.L., Wojcik A., Sohrabi M., Burkart W., Cardis E., Laurier D., Tirmarche M., Hayata I. (2009). Human exposure to high natural background radiation: What can it teach us about radiation risks?. J. Radiol. Prot..

[30] Bartlett D.T. (2004). Radiation protection aspects of the cosmic radiation exposure of aircraft crew. Radiat. Prot. Dosim..

[31] Durante M., Cucinotta F.A. (2008). Heavy ion carcinogenesis and human space exploration. Nat. Rev. Cancer.

[32] Takam R., Bezak E., Marcu L.G., Yeoh E. (2011). Out-of-field neutron and leakage photon exposures and the associated risk of second cancers in high-energy photon radiotherapy: Current status. Radiat. Res..

[33] Yonai S., Furukawa T., Inaniwa T. (2014). Measurement of neutron ambient dose equivalent in carbon-ion radiotherapy with an active scanned delivery system. Radiat. Prot. Dosim..

[34] Staaf E., Brehwens K., Haghdoost S., Pachnerova-Brabcova K., Czub J., Braziewicz J., Nievaart S., Wojcik A. (2012). Characterization of a setup for mixed beams exposure of cells to ^241^Am alpha particles and X-rays. Radiat. Prot. Dosim..

[35] Staaf E., Brehwens K., Haghdoost S., Nievaart S., Pachnerova-Brabcova K., Czub J., Braziewicz J., Wojcik A. (2012). Micronuclei in human peripheral blood lymphocytes exposed to mixed beams of X-rays and alpha particles. Radiat. Environ. Biophys..

[36] Staaf E., Deperas-Kaminska M., Brehwens K., Haghdoost S., Czub J., Wojcik A. (2013). Complex aberrations in lymphocytes exposed to mixed beams of ^241^Am alpha particles and X-rays. Mutat. Res. Genet. Toxicol. Environ. Mutagen..

[37] Sollazzo A., Shakeri-Manesh S., Fotouhi A., Czub J., Haghdoost S., Wojcik A. (2016). Interaction of low and high LET radiation in TK6 cells-mechanistic aspects and significance for radiation protection. J. Radiol. Prot..

[38] Coffman V.C., Wu J.Q. (2012). Counting protein molecules using quantitative fluorescence microscopy. Trends Biochem. Sci..

[39] Dion V., Gasser S.M. (2013). Chromatin movement in the maintenance of genome stability. Cell.

[40] Friedland W., Dingfelder M., Kundrat P., Jacob P. (2011). Track structures, DNA targets and radiation effects in the biophysical Monte Carlo simulation code PARTRAC. Mutat. Res. Fundam. Mol. Mech. Mutagen..

[41] Scherthan H., Hieber L., Braselmann H., Meineke V., Zitzelsberger H. (2008). Accumulation of DSBs in gamma-H2AX domains fuel chromosomal aberrations. Biochem. Biophys. Res. Commun..

[42] Neumaier T., Swenson J., Pham C., Polyzos A., Lo A.T., Yang P., Dyball J., Asaithamby A., Chen D.J., Bissell M.J. (2012). Evidence for formation of DNA repair centers and dose-response nonlinearity in human cells. Proc. Natl. Acad. Sci. USA.

[43] Schmid T.E., Dollinger G., Beisker W., Hable V., Greubel C., Auer S., Mittag A., Tarnok A., Friedl A.A., Molls M. (2010). Differences in the kinetics of gamma-H2AX fluorescence decay after exposure to low and high LET radiation. Int. J. Radiat. Biol..

[44] Dion V., Kalck V., Horigome C., Towbin B.D., Gasser S.M. (2012). Increased mobility of double-strand breaks requires Mec1, Rad9 and the homologous recombination machinery. Nat. Cell Biol..

[45] Cremer T., Cremer M., Hubner B., Strickfaden H., Smeets D., Popken J., Sterr M., Markaki Y., Rippe K., Cremer C. (2015). The 4D nucleome: Evidence for a dynamic nuclear landscape based on co-aligned active and inactive nuclear compartments. FEBS Lett..

[46] Falk M., Lukasova E., Stefancikova L., Baranova E., Falkova I., Jezkova L., Davidkova M., Bacikova A., Vachelova J., Michaelidesova A. (2014). Heterochromatinization associated with cell differentiation as a model to study DNA double strand break induction and repair in the context of higher-order chromatin structure. Appl. Radiat. Isot..

[47] Reindl J., Girst S., Walsh D.W., Greubel C., Schwarz B., Siebenwirth C., Drexler G.A., Friedl A.A., Dollinger G. (2017). Chromatin organization revealed by nanostructure of irradiation induced gammaH2AX, 53BP1 and Rad51 foci. Sci. Rep..

[48] Savage J.R.K., Simpson P.J. (1994). FISH “painting” patterns resulting from complex exchanges. Mutat. Res..

[49] Adhikary A., Becker D., Sevilla M.D., Lund A., Shiotani M. (2014). Electron Spin Resonance of Radicals in Irradiated DNA. Applications of EPR in Radiation Research.

[50] Sevilla M.D., Becker D., Kumar A., Adhikary A. (2016). Gamma and Ion-Beam Irradiation of DNA: Free Radical Mechanisms, Electron Effects, and Radiation Chemical Track Structure. Radiat. Phys. Chem..

[51] Girst S., Hable V., Drexler G.A., Greubel C., Siebenwirth C., Haum M., Friedl A.A., Dollinger G. (2013). Subdiffusion supports joining of correct ends during repair of DNA double-strand breaks. Sci. Rep..

[52] Becker A., Durante M., Taucher-Scholz G., Jakob B. (2014). ATM alters the otherwise robust chromatin mobility at sites of DNA double-strand breaks (DSBs) in human cells. PLoS ONE.

[53] Aten J.A., Stap J., Krawczyk P.M., van Oven C.H., Hoebe R.A., Essers J., Kanaar R. (2004). Dynamics of DNA double-strand breaks revealed by clustering of damaged chromosome domains. Science.

[54] Aten J.A., Kanaar R. (2006). Chromosomal organization: Mingling with the neighbors. PLoS Biol..

[55] Weiss M., Elsner M., Kartberg F., Nilsson T. (2004). Anomalous subdiffusion is a measure for cytoplasmic crowding in living cells. Biophys. J..

[56] Brehwens K., Bajinskis A., Staaf E., Haghdoost S., Cederwall B., Wojcik A. (2012). A new device to expose cells to changing dose rates of ionising radiation. Radiat. Prot. Dosim..

[57] Scherthan H., Adelfalk C. (2011). Live cell imaging of meiotic chromosome dynamics in yeast. Methods Mol. Biol..

[58] Illner D., Scherthan H. (2013). Ionizing irradiation-induced radical stress stalls live meiotic chromosome movements by altering the actin cytoskeleton. Proc. Natl. Acad. Sci. USA.

[59] (2016). R Core Team. R: A Language and Environment for Statistical Computing. https://www.R-project.org.

